# Knowledge, attitudes, and practices regarding malaria transmission and prevention in an indigenous Maijuna community: a qualitative study in the Peruvian Amazon

**DOI:** 10.1186/s12936-024-05121-8

**Published:** 2024-10-18

**Authors:** Kathryn M. Hogan, Michael Gilmore, Graziella P. McCarron, Brian M. Griffiths, Jeffrey W. Koehler, Guillermo A. García, Michael E. von Fricken

**Affiliations:** 1 Department of International Health, Johns Hopkins Bloomberg School of Public Health, Baltimore, MD USA; 2 School of Integrative Studies, George Mason University, 4400 University Drive, Fairfax, VA 22030 USA; 3 The Earth Commons—Georgetown University’s Institute for Environment & Sustainability, 3700 O St. NW, Washington, DC USA; 4 Diagnostic Systems Division, US Army Medical Research Institute of Infectious Diseases, 1425 Porter Street, Fort Detrick, MD 21702 USA; 5 MCD Global Health, 8403 Colesville Road, Suite 320, Silver Spring, MD 20910 USA; 6 Department of Environmental & Global Health, Emerging Pathogens Institute, One Health Center of Excellence, University of Florida, 2055 Mowry Rd, Gainesville, FL 32610 USA

**Keywords:** Malaria, Peruvian Amazon, Prevention, Qualitative research, Indigenous populations

## Abstract

**Background:**

Peru is a low-endemic transmission area for malaria, where the majority (84%) of incident malaria cases are localized to the department of Loreto, which is composed of several geographically isolated rural communities. Recent intervention efforts targeting at-risk Indigenous populations that live in riverine communities in Loreto place emphasis on preventive behaviours to decrease transmission. However, malaria related behaviour change is often dependent upon local knowledge, beliefs, and practices, especially in areas where malaria is viewed an embedded and unavoidable aspect of life.

**Methods:**

This exploratory case study used semi-structured interviews conducted in Spanish between February and March of 2019 to examine the knowledge, attitudes, and practices related to malaria prevention among the Indigenous Maijuna people of Sucusari, Loreto, Peru. Participants who consented were also administered a rapid diagnostic test (RDT) upon the time of interview.

**Results:**

A total of 33 community members were interviewed, and 31 were tested via malaria rapid diagnostic tests, with RDT filter paper subsequently tested using PCR. All test results were negative for malaria. Themes that emerged included: varying knowledge of methods to prevent malaria, reports of observed changes in malaria incidence over time, confusion surrounding malaria transmission, treatment-seeking as a common behaviour, the belief that medications are effective, and the acceptance of bed nets which were viewed as a lifestyle norm.

**Conclusion:**

These shared narratives should be used as a foundation for further studies and health interventions among communities in the Peruvian Amazon with limited access to health services where culturally resonant, community-based health programming is essential to improving health. Takeaways regarding confusion surrounding malaria transmission should also be considered.

**Supplementary Information:**

The online version contains supplementary material available at 10.1186/s12936-024-05121-8.

## Background

Malaria transmission in Latin America has been predominantly localized to the Amazon Basin, where 86% of all cases occur [1]. Within the Amazon Basin, Peru is considered a low-endemic transmission area for malaria, with an estimated 29,745 cases in 2020 [2]. Over the past two decades, malaria incidence in Peru has fluctuated largely due to changes in intervention programs [3]. Following the end of the Global Fund Malaria Project “PAMAFRO” in 2010, cases peaked in 2015 [3]. From 2015 to 2020, case incidence decreased by 76%, likely related to the Peruvian Ministry of Health’s introduction of the Malaria Zero Program (MZP) in 2017 [3, 4]. The MZP targeted regions with a high number of indigenous communities, which have been historically at a higher risk of malaria transmission and took a community-level approach to work towards Peru’s malaria control and elimination goals [4, 5].

Within Peru, 84% of transmission is localized to the Department of Loreto (capital Iquitos), which constitutes nearly a third of Peru’s land mass, but is home to only 3.3% of the Peruvian population [1, 5, 6]. Around a third of the Loreto population live in rural communities, which are often geographically isolated from existing healthcare services [7, 8]. In remote areas that experience low rates of malaria transmission, high mobility of individuals who conduct forest-based economic or subsistence activities outside of their communities such as clearing farmland, hunting, or exchanging goods, can serve as a reintroduction point for transmission, complicating efforts to eliminate malaria [1, 9–11].

The parasite primarily responsible for malaria in the Amazon Basin is *Plasmodium vivax,* occurring at a ratio of 4:1 with *Plasmodium falciparum* [12–14]. *Plasmodium vivax* can be controlled in these communities most effectively by vector control; however, achieving elimination can be a challenge due to its relapsing nature [15–17]. There are several malaria treatment and prevention initiatives directed by the Peruvian Ministry of Health and local non-government organizations (NGOs) that provide free bed nets and anti-malarial drugs, and work to improve regional surveillance, early detection rates, and prompt treatment through test-and-treat programmes [4, 18–21].

Previous studies have examined the uptake of preventive behaviours in the surrounding region [22–24], yet, there is limited information on malaria knowledge, uptake of preventive behaviours, and treatment-seeking behaviours in riverine communities, likely due to logistical challenges in accessing remote populations [22–24]. The studies that exist, are often performed in urban, peri-urban, and rural areas surrounding Iquitos, reported that the acceptability of preventive behaviours by individuals and communities can be affected by factors such as socio-economic status, gender, knowledge of malaria and its mode of transmission, and beliefs about the value, safety, inconvenience and effectiveness of mosquito nets [22, 24, 25]. Reported perceptions included the normalization of malaria symptoms and the idea that malaria is embedded and unavoidable in society, which acts as a major barrier to behaviour change [24]. One study attributed poor uptake of preventive behaviours to a lack of local knowledge about malaria transmission and beliefs that vary by community and household [26].

Successful health interventions in other countries have been shaped around local culture to increase community engagement and effectively communicate intervention objectives [27, 28]. In Loreto, one such successful intervention implemented is the widespread use of insecticide-treated nets (ITNs), with a reported ITN ownership of over 98.7% of households [29]. ITN use may have been successful because it incorporated the intervention into the community’s cultural background, where the use of ITNs became habitual and behaviours were passed down over generations [29].

To better understand malaria-related behaviours among high-risk populations living in remote riverine communities, this study aimed to: (1) explore local attitudes and knowledge specific to transmission, treatment-seeking, and prevention of malaria among the Maijuna community in Sucusari; (2) understand how knowledge and attitudes toward malaria impact the behaviour of community members, including uptake of preventive actions, treatment-seeking, and malaria care; (3) understand barriers to uptake of preventive behaviour such as sleeping under bed nets and participating in indoor residual spraying (IRS) among community members.

## Methods

### Study site

This study was conducted in collaboration with the Maijuna (Orejón) Indigenous group of the northeastern Peruvian Amazon. The Maijuna are a Western Tucanoan people with a population of approximately 600 individuals, making them one of the smaller and more vulnerable indigenous groups in Peru [30]. The Maijuna live in four communities in the department of Loreto: Puerto Huamán and Nueva Vida along the Yanayacu River, Sucusari along the Sucusari River, and San Pablo de Totolla along the Algodón River [30].

**Fig. 1:**
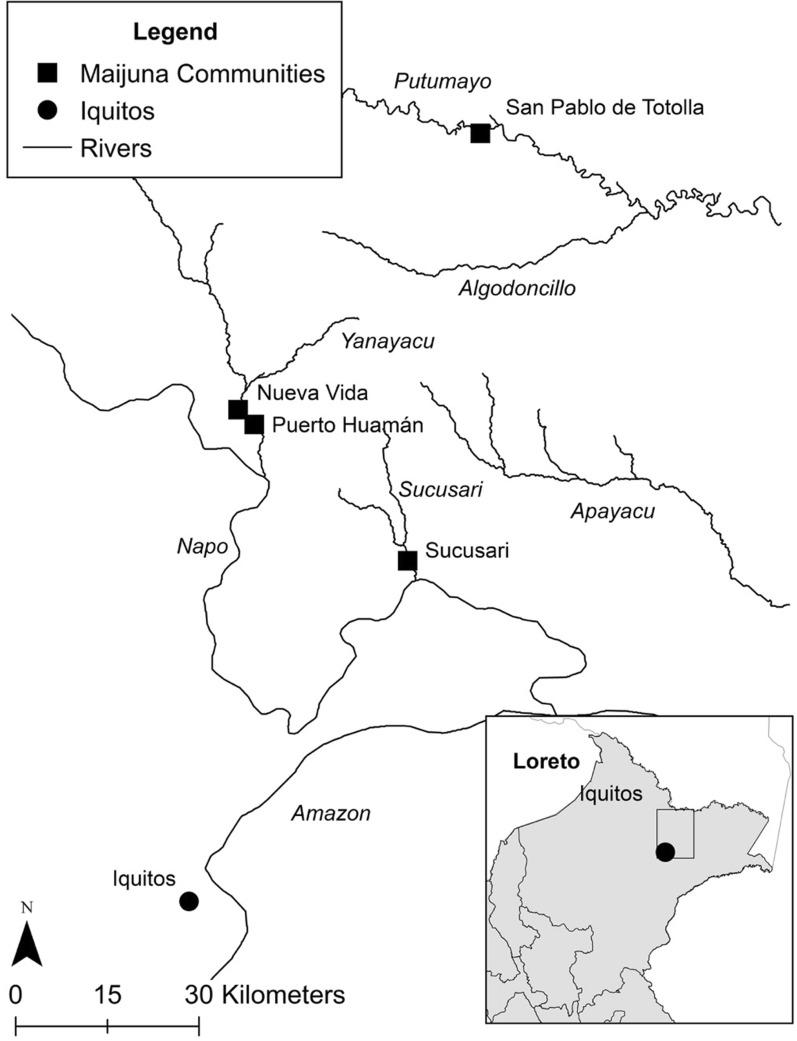
Map of the study site, the Maijuna community of Sucusari in the northeastern Peruvian Amazon

Interviews were conducted in Sucusari (Fig. 1), located approximately 126 km by river from Iquitos in the Sucusari River basin, a tributary of the Napo River. The nearby health posts and hospitals commonly visited by individuals living in Sucusari are located in Puinahua which is approximately 12 km away, Isla Tamanco which is 20 km away, and Mazán which is 40 km away. This region is characterized by a mean annual temperature between 27 and 29 °C, and it is warmest between September and October, with relative humidity ranging between 87 and 93% [31]. Malaria prevalence in Loreto varies seasonally with rainfall and water levels, with high rates of transmission typically occurring 2 months after the river level rises to its peak, and low rates of transmission typically occurring 2 months after the river levels are lowest [31].

### Sample population

The population of Sucusari is 172 people among 32 monofamilial or plurifamilial houses [32]. Roughly half of the individuals living in Sucusari are Indigenous Maijuna, while the other half identify as *mestizos*, or mixed Amerindian and Iberian descent [32, 33]. Sucusari represents one of the many riverine communities in the Peruvian Amazon that are located over an hour boat ride from the nearest health post, and whose residents’ subsistence strategies require frequent visits into the rainforest [30]. The community’s acting health promotor was also interviewed and given the pseudonym of Martín for this study.

### Study design

This study was designed as an exploratory case study [34], to provide meaning and understanding to experiences in a given context and is characterized by an absence of preliminary research or hypotheses [35, 36]. An exploratory case study provides context to multiple bounded systems, in this case, members of the community of Sucusari [22, 24, 34]. As a result from comments made by community members about malaria in Sucusari, this study was designed to examine the perceived burden of malaria within the community and the types of resources available to prevent and treat infection.

Feedback on study design was sought in advance with community leaders, allowing them the opportunity to provide comment, after which consent was given to introduce the study to all community members, allowing for public comment and questions. Given that Indigenous communities are vulnerable populations due to historical social marginalization and exploitation, care was taken to conduct this study ethically following the principles of respect for persons, beneficence, and justice [37]. This study was designed to provide results that could be used to strengthen community-based public health intervention strategies, encourage adoption of preventive behaviour, and promote a general understanding of malaria transmission [38]. Each person was asked to participate in an interview and/or a malaria RDT, and were audio recorded after receiving Prior Informed Consent. There were no incentives to enroll in this study, other than to share information that could be disseminated more broadly to improve public health, and participants could opt out of an interview at any time during the process.

Several steps were taken across all stages of this project, from the creation of research questions through the analysis stage, to maximize the appropriateness and trustworthiness of results and limit the impact of bias. Recording of interviews and field notes on observations during interviews were used to ensure descriptive validity and the factual accuracy of what the researchers report [39]. Purposive and iterative sampling were used to ensure that experiences shared during interviews represented those of the community, and that participants included represented multiple age groups, different gender perspectives, and those who were and were not ethnically Maijuna [39]. Interpretive validity, which focuses on understanding the meaning and intentions of reported events and behaviours, was addressed by spending prolonged time in the community to understand patterns of behaviour, as well as through the inclusion of several questions about why a participant behaved in a specific way, or how they felt about a specific event occurring [39].

### Data collection, transcription, and analysis

Semi-structured interviews [24] were conducted in-person in Spanish, to assess knowledge, attitudes, and practices of Sucusari community members related to malaria. Rapid Diagnostic Tests (RDTs) (*CareStart*™ Malaria RDTs HRP2/pLDH (Pf/Pv)) were also performed to determine point prevalence in the community. Nucleic acid from thirty RDTs were extracted using the EZ1 Virus Mini Kit 2.0 (Qiagen) and the EZ1 Advanced XL robot (Qiagen) and tested using a *Plasmodium* spp. real-time PCR assay [40] and a flavivirus spp. real-time RT-PCR [41]. The RDT test strip was removed and added to a microfuge tube containing 500 uL ATL buffer, heated for 15 min at 56 °C, and 400 uL extracted with a 60 uL elution volume. Samples were tested in singlet for RNase P (human sample control) using TaqPath (ThermoFischer Scientific) to confirm sample integrity and efficient nucleic acid extraction. Extracted nucleic acid (5 ml) were tested on the QuantStudio DX real-time PCR instrument (ThermoFischer Scientific) using a *Plasmodium* spp. real-time RT-PCR (SuperScript II/Platinum taq from ThermoFischer; cycling: 95˚C × 5 min; 45 cycles (95 °C × 1 s, 60 °C × 20 s); 40 °C × 30 s) and a flavivirus spp. real-time RT-PCR assay (TaqPath 1-Step RT-qPCR Master Mix, CG from ThermoFischer; cycling: 50 °C × 15 min; 95 °C × 5 min; 45 cycles (95 °C × 5 s, 55 °C × 20 s). A positive test result required a Cq value of < 40.

Purposeful and snowball sampling methods were used to select participants to ensure that diverse and information-rich perspectives were considered [42]. Members of every family who were 18 years or older, currently residing in Sucusari, and who spoke Spanish were also welcomed to participate in interviews to ensure inclusion of all who wished to share perspectives. Interviews (see Supplementary Information) were locally adapted and informed by published survey instruments recently used in qualitative studies related to mosquito-borne diseases in Peru [24, 42]. Three pilot interviews were conducted in February 2019 with members of the community to ensure clarity and acceptability by participants [24]. The final interview guide was deployed for all interviews conducted by the same two researchers in the study period between February and March 2019.

In the interviews, participants were asked about their sociodemographic information, health challenges in the community, knowledge of malaria, behaviours related to malaria, attitudes on encounters with health programmers or visiting doctors, and experiences having malaria, if applicable (See Supplementary Information). Interviews lasted between 20 and 90 min. All interviews were recorded and transcribed, with field notes taken by both researchers after each interview summarizing main points. All interviews were translated from Spanish to English by a researcher team member present during interviews. Transcriptions of interviews were coded following qualitative coding methods outlined by Saldana [43]. The themes that emerged through this process were then categorized into the knowledge, attitudes, and practices framework to align with the research questions (Supplementary file 1).

## Results

Thirty-three participants agreed to be interviewed (Table 1), 27 of whom volunteered to have an RDT administered as well. Four community members did not wish to participate in an interview but volunteered to have an RDT done. There were no positive test results for malaria using the RDTs or reported in the community during the study period. Testing of all 30 RDTs resulted in a positive human RNase P test result, indicating the integrity of the blood sample as well as efficient nucleic acid extraction. All 31 RDTs tested negative for *Plasmodium* spp. and flavivirus spp. by real-time RT-PCR.

**Table 1:** Demographics of participants in interviews of knowledge, attitudes, and practices surrounding malaria in the Maijuna community of Sucusari in the Peruvian Amazon

Variables	N = 33 (%)
Gender	
Male	23 (69.7)
Female	10 (30.3)
Ethnicity	
Maijuna	17 (51.5)
Non-Maijuna	16 (48.5)
Age	
18–24	5 (15.2)
25–34	9 (27.3)
35–44	6 (18.2)
45–54	6 (18.2)
55–64	3 (9.1)
65–74	4 (12.1)
Highest level of education	
None completed	1 (3.0)
Some primary	11 (33.3)
Completed primary	9 (27.3)
Some secondary	7 (21.2)
Completed secondary	2 (6.1)
Some university	1 (3.0)
Unknown	2 (6.1)

Ten women and 23 men participated in interviews, with ages ranging from 18 to 74 years old. There was a difference in the average reported number of malaria cases participants experienced in their lifetimes between males and females, with 23 male men respondents reporting they had malaria an average of six times and six women reporting they had malaria an average of four times in their lifetimes.

### Themes

The perspectives and themes that emerged during interviews were grouped as sub-themes within the main knowledge, attitudes, and practices framework (Table 2). There were no observed differences in themes that emerged by gender. Additionally, there were no observed differences in themes that emerged among those who were ethnically Maijuna versus participants who were not ethnically Maijuna.

**Table 2:** Major themes and sub-themes of interview responses of knowledge, attitudes, and practices of community members in Sucusari, Peru, towards malaria

Theme	Sub-theme
Knowledge	Confusion about how malaria is transmitted
	Focus on the differentiation of malaria species
	Varying understandings of behaviours to prevent malaria
Attitudes	Observed change in incidence of malaria over time
	Belief that health worker visits decreased malaria in Sucusari
Practices	Treatment seeking for malaria is common
	Participants adopted several prevention and treatment behaviours

### Participant knowledge about malaria

#### Confusion about how malaria is transmitted

The most prominent theme involved the community’s knowledge of malaria transmission. All but three participants referenced the role of mosquitoes in transmission as represented below:

*“They say it comes from the mosquitoes, sometimes from water when you leave little pots outside and they fill with water, the mosquitoes breed there and then they bite you and you have malaria.”* [P01]

*“Malaria comes from when you throw out your tins and they fill with water and that’s where the mosquitoes lay eggs. That’s how the mosquitoes grow and raise their young, then every time a mosquito bites you, you can get malaria.”* [P02]

However, there was variation in the specific mechanism of malaria transmission from mosquitoes that was described. Modes of transmission described included the bite of an infected mosquito, drinking contaminated water where mosquitoes reproduce, and drinking any contaminated water. Two participants associated malaria with a negative spiritual force. One participant listed three different modes of transmission throughout the course of one interview:

*“If a mosquito bites you and it has malaria then it bites me then I have malaria too. Then they told us that malaria was not going to go away if there was not clean water.”* [P03]

*“They say you shouldn’t eat meat from the forest like from huangana* [white-lipped peccary (Tayassu pecari)] *or sajino* [collared peccary (Pecari tajacu)] *or sachavaca* [tapir (Tapirus terrestris)] *because then you always get malaria.”* [P03]

*“But sometimes we go upriver, and for example I go to Belisario, and then you drink water from the forest, and I came back with malaria.”* [P03]

Some community members attributed malaria transmission to specific locations, linking malaria to the remote environment they worked in when they were a logger. When asked follow-up questions, participants frequently did not specify why they thought that those locations had more malaria than the community, but some attributed this difference to drinking unclean water in those locations. These beliefs are reflected below:

*“Sometimes when we leave for other parts, we get it.”* [P04]

*“They say [malaria comes] from mosquitoes, from water, and we don’t know sometimes…I believe more in the water. Because sometimes when I went to the forest with my brother-in-law, we drank water from the aguajales* (swamps) *and in the afternoon we had symptoms of malaria.”* [P05]

*“Malaria comes from the water. Now everyone drinks clean water and there is no malaria. If you do not drink clean water everyone gets sick. Nobody can escape. Here, there are loads of aguajales,* (swamps) *hundreds of them. When it is winter, the aguajales have water and the mosquitoes are there. And what does the water there do? It gets yellow, like rotten water. The mosquitoes live there, and there is the malaria. In winter when it rains, all of that water washes out and passes here, and when you drink that it causes harm and malaria. Diarrhea also comes from that.”* [P06]

More than half of participants emphasized that malaria transmission was highest after the river rose. The remaining participants said that malaria incidence remained the same throughout the year. Both perspectives are expressed below:

*“Yes, malaria always comes back when the floods come. For instance, last year when it flooded there were some cases. In July and August. Because the water has started to go down, but it stays in the lakes and the ponds. That’s where malaria comes from. There are not many cases, but there are cases. This is every year, after the water comes. But when there are no floods there is no malaria* [P07]

*“No, it does not have exact times, it can get you any time.”* [P08]

When asked where malaria comes from, many community members used phrases such as “they say” or “they tell us” before sharing their perspective. When asked when and where they first learned about malaria, every participant responded that they first learned about malaria when they themselves were sick, or when they witnessed their family member get malaria. They frequently learned about the disease from the health practitioners who were treating them or their families, but some participants also referenced health workers or doctors who visited and provided information on malaria.

#### Focus on the differentiation of malaria species

When participants were asked to relate what they knew about malaria, 18 (54.5%) named the different types of malaria common to the region, including *P. vivax* and *P. falciparum.* Additionally, participants often identified which type of malaria they had when discussing their own experiences, differentiating symptoms, severity, mortality, and timeline between them:

*“If you say that you are shaking, they gave you the three pills for vivax. If you say that your whole body is hurting, they give you the pills for falciparum. But when they give you the wrong pills it does not do anything.”* [P03]

*“Vivax gives you a headache, then you get shivers. You shake. When you shake a lot, you get cold, you get a fever. Then you know it is vivax. Falciparum, your body hurts, your eyes, your bones, everything. Then you know it is falciparum. It gives you a fever too. When it is more grave it makes you vomit, you don’t feel like eating”.* [P04]

Three participants specified a third type of malaria as *maligna* (evil). While all agreed that this type caused the most severe symptoms, two of these participants referred to *maligna* in place of *P. falciparum*, while one participant referred to it as a separate species discussed below:

*“One is vivax, the other is falciparum, and the third is maligna. This is the strongest one that you can see here – maligna. Because it is a diabolical spirit.”* [P08]

#### Varying understandings of behaviours to prevent malaria prevention

Some respondents did not know or remember specific ways to prevent malaria, but many described methods that they believed were effective. The most common response was to go to bed earlier and leave bed later, although the majority who identified this also indicated that they did not typically follow this:

*“Yes, to prevent malaria you have to go to your bed at 5:30 pm, wear long sleeves, long pants with boots. But people here are not used to that so they do not do it.”* [P09]

*“Lots of people say you can sleep early because at 6 the mosquitoes come, that’s what the doctor says. I do not do that.”* [P10]

Participants also discussed bednet use as a commonly adopted behaviour to prevent malaria. They linked going to bed earlier with the protection from mosquito bites that the nets provide. They also specified the use of ITNs, with insecticides to kill mosquitoes:

*“You can use your mosquito net. They have poison on them that kill mosquitoes.” [P04].*

When asked about other ways to prevent malaria beyond sleeping under bed nets, the lack of consensus about the transmission process reemerged, as a small portion of participants indicated that drinking clean water could keep them from getting malaria:

*“To prevent these diseases, you have to drink clean water.”* [P06]

*“Now that we drink clean rainwater, we don’t get it too often”* [P11]

Nearly half of participants emphasized the importance of emptying containers with water to decrease the number of mosquito breeding sites. They indicated that health workers who they saw when sick and workers who visited Sucusari from other locations for testing, treatment, and programming shared this information:

*“Take care of yourself. Don’t let mosquitoes bite you. Don’t have containers outside the house where mosquitoes can lay eggs.”* [P12]

*“They tell you not to toss out trash that can collect water, they say that the mosquitoes reproduce inside there.”* [P13]

The health promotor and schoolteachers in Sucusari discussed ways to prevent malaria which they learned while attending separate trainings and workshops provided by the Peruvian government and local NGOs. Below, one of the teachers in Sucusari shares what the workshops taught:

*“The teachers used to participate in workshops to learn about malaria so that we can teach the children to prevent malaria as well. Do things like go to bed early, not keep containers of water around, don’t toss trash outside because malaria can reproduce there, in containers of water they lay their eggs and reproduce.”* [P14]

### Participant attitudes about malaria

#### Observed change in incidence of malaria over time

The most prominent theme in the attitudes of participants towards malaria was regarding their experiences of the change in malaria incidence over time, and their perceived reasons for this change. All but one participant mentioned that incidence had decreased in the community recently, after a long period of increased cases. Some referred to a time before their families knew what malaria was:

*“Before, my grandparents were dying in large quantities. They didn’t know what was killing them. At that time, there wasn’t any medicine.”* [P15]

Respondents attributed the recent decrease in incidence to many causes, including medicine, fumigation, drinking clean water, having a health promotor in the community, new health facilities nearby, and health worker visits to the community with tests and medications. When asked about how malaria has changed over time, many participants explained how the community was previously filled with cases of malaria:

*“It has decreased. It has gone down a lot. But there was a lot in 2013-2014. The people here were all sick, truly. Everyone had it, kids, old people, everyone. And it was daily that people had malaria. This person has it, this person is going to the health post with malaria… and, up to now, we don’t know, if the malaria has left, if it got tired of us, we don’t know… there is malaria, but not like before.”* [P05]

*“There were times when the entire community, no lie, maybe except three or four people, had malaria. I say it comes from the water because we would drink water directly from the river.”* [P06]

*“We have medicine and treatment here, and close by in the health post that we can take to get better faster.”* [P16]

Participants were asked if they thought that there would be a time when malaria would not exist. There were mixed responses to this question, and some participants indicated that if the health promotors, doctors, and fumigators continued to visit, then malaria might end:

*“If there is more support for us from the government, in the future for our children, I believe that sometime in the future we don’t have to suffer from malaria.”* [P17]

*“This depends on the health center, if they keep combating the mosquitoes, fumigating and sending treatment. If they do, then the malaria could be finished.”* [P13]

While those above felt it was the government’s responsibility to eliminate malaria, some agreed that malaria elimination may be possible in the future, but felt that individuals also need to take responsibility to stop malaria:

*“Well for that I say it all depends on us because if we do not maintain our clean environment, we will get malaria again and the fevers will start again.”* [P14]

Others who did not believe malaria would ever go away explained that mosquitoes would never go away completely, and, therefore, malaria would always be around:

*“It is going to keep coming because it can always come from another community. One person travels and gets infected, they come back here and a mosquito bites them and then bites me, and now I have malaria too.”* [P1]

#### Belief that health worker visits decreased malaria

There was an overall attitude throughout interviews that visits by health workers were instrumental to the decreasing number of malaria cases. Participants described a health brigade from the local health center that came to Sucusari for frequent check-ins when the number of malaria cases rapidly increased. Many participants explained that they would visit and test the whole community during an outbreak, provide medications to those who were infected, and return later to re-test:

*“In the times when there was lots of malaria, they came to build capacity to make sure there weren’t any containers of water open that fill with rain and then stay there. But this stopped because now [malaria] doesn’t exist.”* [P08]

*“The brigade of doctors came from Mazán to do the tests, but we didn’t feel anything I felt healthy. But we all came out with malaria, the disease was living inside of us.”* [P16]

Community members also shared that health workers came to provide educational programming, distribute bed nets, and perform IRS in the community. Many interviewees referenced a health worker delivering bed nets within the past year, however, the times and frequencies for other visits varied more across all interviews:

*“There’s always workshops that the health workers put on in the community if there is a new disease or on malaria*.” [P12]

*“The health worker from Mazán always used to come and he would explain what the symptoms of malaria are.”* [P07]

The overall attitudes towards these visitors were positive, and, as discussed above, many of the interviewees attributed the decrease in malaria to their visits. When asked if anyone came to the community, and about their impression of those who had, participants responded as follows:

*“It was good, because they came here, and we did not have to spend the gas and time to go to Mazán. I think the brigade did good work because they kept coming and over time malaria has decreased. Now, we have a health post close too.”* [P13]

*“It [malaria] went down when the fumigators came. One big drop.”* [P15]

*“Good, it [fumigation] killed all of the insects. But it burned everyone’s faces. You couldn’t lean against the house. But all of the mosquitoes and cockroaches died in the house.”* [P19]

Despite the overall positive attitude toward fumigation, all participants who discussed it specified that fumigations have not occurred since around 2016 after cases of malaria decreased. Some participants also mentioned that the health promotor can ask the government for fumigators to visit again and specified why they have not continued to spray:

*“Now that there is not much malaria they do not come. But the health promotor has the responsibility to go to the government once per year to ask them to come and here they don’t do that.”* [P17]

*“After they saw that there wasn’t much malaria in the community anymore, they stopped coming. Since a year ago. They can send a request for fumigation and they come.”* [P20]

Six community members clarified that the community does not ask for fumigations anymore because previous fumigations killed bees that were being raised as part of a community-based bee-keeping project:

*“Now we don’t want them to fumigate because we are raising bees. Last time they fumigated all of the bees ran off. I lost four hives of bees last time.”* [P03]

### Participant practices related to malaria

#### Treatment-seeking for malaria is common

The most prominent theme describing practices related to malaria was a person’s experience seeking treatment and care. Every participant reported having malaria at least once. When asked to describe both their first and most recent experiences having malaria, every person interviewed except for the youngest participant, who was 18 years old, reported visiting a health center for at least one of their cases. The one person who did not visit a health center was tested and treated by the health promotor. Several other participants also reported visiting the health promotor instead of, or before, visiting the health post during their most recent experiences with malaria symptoms. Fewer participants reported being diagnosed and receiving treatment when the brigade visited Sucusari and tested each member of the community:

*“The brigade of doctors from Mazán came and took my blood and it came out with malaria vivax. Then they gave me medicine.”* [P16]

One community member indicated that the severity of their symptoms progressed as they waited for their test results from the brigade. Others indicated they waited to seek treatment until their symptoms worsened because they thought it was a different sickness that would pass, they were waiting for transportation or money for transportation, or were away from the community:

*“[I waited] one week. That’s why I almost died, because they didn’t come quickly. We had to go to Mazán. The post in Puinahua didn’t exist yet, it is new.”* [P15]

For many of the participants’ first cases, which were often before the health promotor position had tests and medications in the community, there were more barriers to seeking treatment, often related to transportation or financial issues:

*“There wasn’t any money. We couldn’t transport ourselves, [or] buy gasoline.”* [P15]

*“We were in the forest, and because the river was low, it took a long time to get back.”* [P01]

*“I got it on a river outside of Mazán when I was up there with the loggers. It took us 15 days to get out and get to the health post in Mazán.”* [P21]

Interviewees reported visiting three health posts near Sucusari, in Puinahua, Mazán, and Tamanco, to seek treatment. When the health promotor was given RDTs and malaria medications by the government, this provided another option for treatment-seeking. Some participants discussed their considerations when deciding on which health post to visit. This decision appeared to vary over time, based on which health posts were open and accessible:

*“If we want to know faster, we can go directly to Mazán. We went to Puinahua with symptoms of malaria, then they did the test, then they send the test to Mazán and after 3 or 4 days you get your results. So Mazán is a higher expense but your results are immediate.”* [P08]

*“Well, if we go to the health post we will have to wait in line for a long time, let’s go to Martín’s [health promotor] house and see if he has the tests.”* [P22]

Interviewees were also asked about how long they waited after the onset of symptoms before seeking care. Participants typically waited longer during their first time with malaria than their most recent time because, initially, they did not know what it was. On average, the participants also waited less time to seek treatment for their children than they did for themselves, waiting an average of 3 days before taking their children versus nearly a week for their own cases.

#### Participants adopted several prevention and treatment behaviours

Since every participant had malaria at least once, they also shared their behaviours related to prevention and treatment. Nearly all participants stated that medication was effective at treating malaria. Participants emphasized the importance of taking medicine, receiving the correct medicine for the specific species of malaria, and completing the full dosage. They attributed their recovery to the medications. When asked if they finished their full treatment and why, participants responded with the following statements on their experiences with taking medications for malaria:

*“Once you finish your treatment you recover fast.”* [P23]

*“Yes, because I wanted to get healthy.”* [P12]

*“If you don’t finish, you’re going to go back to the health post because you are not going to get better.”* [P08]

All participants who said they did not finish the full course of treatment acknowledged that they knew they had to complete the full dose to get rid of the parasite, but they either thought they were recovered, thought the pills were too bitter, or felt too sick or weak from the pills to complete:

*“Lots of people arrive but then don’t take their whole treatment and they get malaria again. I am telling you these things, but sometimes I don’t take my complete treatment either. Then I got malaria again. Sometimes I take 4 or 5 pills and I feel healthy, so I stop taking them.”* [P05]

*“The pills are really strong; they make you dizzy and you walk like you are crazy.”* [P09]

Participants were also asked about their use of traditional remedies and how their parents previously treated malaria. There was a wealth of knowledge surrounding traditional remedies which varied by age groups. Traditional remedies discussed included grinding, boiling, and drinking the resultant liquid made from one of many possible ingredients, including the shell of the yellow-footed tortoise (*Chelonoidis denticulata,* local name *motelo*), the bark of the *remo caspi* tree (*Aspidosperma* spp.), *clavo huasca*, the roots of the *huasai* tree (*Euterpe precatoria*), and the bark of the *abuta* vine (*Cissampelos pareira*). Even though participants were familiar with traditional remedies to treat malaria, those who mentioned different remedies all preferred to take medication when it was accessible:

*“You wouldn’t drink that here because you’re close [to the health post] but when you’re out in the forest and logging, yes. This is what your parents teach you, from your grandparents.”* [P05]

*“They [parents] used this [traditional remedies] to cure themselves. But when you take it, it doesn’t cure anything. One minute it would pass, then come back every bit.”* [P15]

*“Here, there are lots of plants that are medicines that they say you can take to get better, but you really have to go to the health post.”* [P17]

There was also an association made during many interviews between the bitterness of the medications and the bitterness of traditional remedies such as those made from *abuta* and *remo caspi*. The association indicates that some community members believed the characteristic of bitterness was related to the effectiveness of the traditional remedies and/or medications:

*“There is a vine that’s called abuta, it’s very bitter. You cook it because the malaria pills are also very bitter, it’s the same as the vine.”* [P07]

*“Well, sometimes when you don’t finish the pills and you get it again lots of people say that medicine from the abuta plant, it is really bitter, and it leaves your body clean and is good to stop malaria.”* [P14]

Participants also discussed the common practice of nightly bed net use to prevent malaria. Every participant reported using a bed net each night, and the majority had used them for their entire lives. There were two families that more recently introduced this behaviour into their routines when health workers provided everyone in the community with free nets:

*“No you have to sleep with one because how are you going to sleep outside with the mosquitoes”* [P01]

*“The government donated some to us, but before that we bought them.”* [P16]

Common complaints about the nets provided by the government were that the mesh was too big to keep out mosquitoes or that they were too hot or cold depending on weather. Some community members showed us a different, thicker type of net with smaller holes that they purchased themselves, and typically used when it was colder. Additionally, the nets provided by the government, which were treated with insecticides, often caused rashes and burns on the skin. The nets were sometimes washed or aired out before use to remove the insecticide and avoid irritation:

*“I like them, but sometimes at first they burn our faces. You have to wash it first and get all the poison out.”* [P24]

*“When it’s new it burns your face.* [What do you do so it doesn’t burn you?] *You hang it up and don’t sleep under it for a while. Put it outside, so that the mosquitoes smell it.”* [P12]

### Summary of results

The results from the 33 semi-structured interviews with community members provided an understanding of the knowledge, attitudes, and practices related to malaria in the Maijuna community of Sucusari. Participants indicated that, despite several visits from health workers, individual trips to health posts to treat malaria, and having a trained health promotor in Sucusari who can test and treat malaria, there is still confusion about how it is transmitted. This confusion appeared to have minimal impact on preventive practices like ITN use each night, which is an accepted practice and embedded in the lifestyles of nearly all community members. Affirmative attitudes towards the effectiveness of anti-malarial medications were consistent and the need to complete the full course of medication to treat malaria was common, but not always practiced. Barriers to seeking treatment were often distance, money for the transportation, or waiting to see if the symptoms were caused by something other than malaria. The belief that malaria would decrease or be eliminated in the future was common, but many participants stated that this outcome would rely upon continued outreach from the government as well as individuals within the community caring for themselves and their families.

### Study limitations

One limitation of this study was that interviews were translated and transcribed into English, which presents the possibility that some of the narrative and experiences of interviewees were not fully conveyed [44]. Additionally, while the interviewers spent 6 months and 1 year living in the community prior to the study, they were raised within the United States, each with perspectives shaped by Western views of science and medicine, which differ from those of the participants. Being external to the community likely limited the researchers’ ability to interpret the local context and responses of participants.

Another limiting factor was that more men than women volunteered to participate in interviews, which reflected the larger proportion of men than women in the community, as well as women being less interested in participating in the interviews. During interviews, all adult household members were asked if they wished to participate, and the ratio of males and females included in the study was similar to the proportion of adult males to adult females in the community.

A final limitation is that studies investigating knowledge, attitudes, and practices often include a quantitative framework. However, this possibility was not feasible for this study due to the small population of the community. Additionally, the exploratory nature of this study lent itself to qualitative methodology, which aimed to understand community themes surrounding malaria in depth, rather than test a known hypothesis. Therefore, the results should be used to inform adjustments to health programming and further studies with a broader scope.

## Discussion

This exploratory case study examined the knowledge, attitudes, and practices surrounding malaria transmission, prevention, and treatment seeking in Sucusari, a riverine community in the Peruvian Amazon. Semi-structured interviews with community members provided information on how previous health programming resonated with the community, how local knowledge and attitudes impacted their behaviours related to malaria prevention and treatment seeking, and identified gaps in understanding about malaria transmission to inform future health programming. Each major theme (knowledge, attitudes, and practices) contained several sub-themes (Table 2).

The understanding of the source of malaria and transmission varied throughout the community. While 26 (78.8%) participants reported that malaria transmission was in some way related to mosquitoes, there were 15 (45.5%) who also mentioned that contaminated water or water with mosquito eggs in it could cause malaria. This confusion has been reported in other studies, dating back decades to a survey in the Philippines with similar reports of transmission being caused by germs in contaminated water, as well as by mosquitoes [45]. These results suggest that previous community-based health programming may not have resonated or reached all intended community members. The confusion surrounding the role that clean water plays in transmission could also be due to the timing of health interventions and educational programming on water, hygiene, and sanitation that occurred at similar times in Sucusari. Several participants indicated that malaria incidence decreased when they began drinking clean water. This suggests that perhaps heath workers came to educate the community on malaria around the same time when water filtration systems were first introduced, making it difficult for community members to differentiate between the interventions. Alternatively, many health programs encourage emptying containers that might collect rainwater to decrease potential mosquito breeding environments and decrease the risk of malaria. This could be the reason that 17 participants mentioned that emptying containers filled with water is important to avoid drinking this water and/or to decrease the numbers of mosquitoes in the area. Other studies from the Amazon have also reported confusion surrounding how malaria is transmitted [22, 24]. It is vital for health workers to understand a community’s exposure to other health programming, and the local context and realities before presenting educational programing, as the malaria transmission cycle is quite complex and could appear to be caused by several phenomena [46].

Confusion about malaria transmission and subsequent differences in knowledge of preventive practices were not directly related to the uptake of preventive behaviours. Everyone in the community reported sleeping under a bed net, and for most this was a behaviour passed down from the previous generation. Additionally, some participants used the same Spanish word, *cama*, to describe their beds and bed nets, suggesting that the use of bed nets is deeply rooted in their culture, as it is in many other communities in the Peruvian Amazon [22, 24]. Since some participants report buying their own nets, and switching these with the government-provided nets depending on weather, it is important to note that while they do practice this behaviour, it is potentially less effective than if they used the ITNs [47]. Previous studies have also suggested that changes in mosquito biting times present barriers to the effectiveness of ITNs, as it would decrease protected time spent under bed nets [48]. However, based on the participant responses that people very rarely go to bed early to avoid peak mosquito biting times, their risk may not change even if the mosquito biting times do shift, since these individuals were already exposed.

The theme of malaria species differentiation emerged as a common topic, likely because the RDT that the health promotor in Sucusari used, and malaria treatments, differ by species. Community members often discussed their experiences having both types of malaria, even though only 25% of malaria cases in the region are typically *P. falciparum* [13]*.* This could be attributed to characteristics specific to *P. vivax,* such as how it frequently causes latent, asymptomatic phases and reoccurrence of malaria [49]. If community members experienced these latent, asymptomatic phases, they would be less likely to seek treatment and, therefore, might report experiences of *P. vivax* less. This explanation also aligns with the reports that community members were diagnosed with *P. vivax* when health workers tested everyone in Sucusari, even those without symptoms. Alternatively, these reports could be due to participant recall bias, because if participants had more severe symptoms during their experience with *P. falciparum,* which is typical*,* they may have been more likely to remember the experience and report it years later.

Results surrounding treatment seeking behaviours showed that seeking treatment and completing medication were perceived as necessary to combatting malaria. When asked if any traditional remedies existed, most participants discussed the use of the same plants and tortoise shell. Many indicated that they learned about the remedies from their parents or others in the community. However, when asked how their parents treated malaria, the answer was often that their parents did not know what malaria was, and they died from it. Suggesting that the remedies may have been used more broadly to treat the symptoms associated with malaria, such as fever and chills. This possibility also aligns with how some participants reported the use of these remedies to ease symptoms until you get to the health post. Interestingly, no one reported preference for traditional remedies over medications, despite having knowledge of them, which reiterates the positive attitude towards anti-malarial medication. Participants frequently connected the bitterness of pills to the bitterness of traditional remedies, which potentially contributed to the acceptability and adoption of medications.

The theme that malaria incidence has decreased since a surge of cases in 2014 was largely attributed to the health worker visits. This suggests that despite confusion surrounding the cause of malaria, community members positively perceive visiting health workers and the malaria interventions they bring. Additionally, there appears to be a transition occurring from the belief that malaria is inevitable, which was recently discussed in a study in Peru [24], to a belief that malaria could end in the future. This is reflected in participants’ statements describing their parents, who died of malaria because they did not have medication, to then saying that an end to malaria “depends on us” and “depends on the health centre.” This suggests that health programming in Sucusari is working, despite some identified gaps, and should continue to serve and support this community.

These findings can be used to inform future health programming related to malaria in the region, such as targeted education campaigns to address knowledge gaps that persist even with ongoing malaria health interventions in the region. The experiences of community members who participated in this study, along with the malaria case numbers in Loreto, indicate that efforts to eliminate malaria in the region are progressing. However, total malaria elimination in the Peruvian Amazon will be challenging and will require ongoing engagement from local, regional, and national government stakeholders with high risk communities to continue decreasing case incidence. Additionally, while dengue virus was not specifically discussed during interviews, this study’s findings may be relevant for prevention efforts given the expansion of dengue virus across Peru in recent years [50]. Particularly, the results on knowledge and attitudes towards insecticide spraying and elimination of mosquito breeding sites are relevant for *Aedes aegypti* mosquitoes that transmit dengue viruses as these interventions are part of the integrated surveillance and response strategies [50].

The results of this study also show that there are gaps in the health communication that community members receive, which results in confusion about how malaria is transmitted. The gaps could trigger more precise guidance that emphasizes the source and transmission mechanisms of malaria and highlight preventive behaviours, using visual aids and culturally relevant materials to ensure the information is accessible and easily understood by community members. This study also identifies the key role that the health promotors and health posts play in malaria control, and highlights the importance of engaging these local health promotors and leaders in the design and delivery of health messages that resonate with individuals. Future health programming in communities should target health leaders responsible for disseminating information to the larger community, including health promotors and health post workers, with training that ensures provider communication that is respectful and thorough.

In addition to informing future practices related to malaria control in the Loreto region, the results from this study also emphasize the importance of continued qualitative malaria research among communities in the Amazon Basin living in high-risk areas. As the landscape of malaria changes and efforts become more targeted, an understanding of the perspectives of communities such as Sucusari will become increasingly important. It is crucial to pair quantitative studies regarding malaria with studies that provide the perspective and depth of those experiencing malaria, who will be vital to malaria elimination in the region.

## Conclusion

Despite decreasing global incidence of malaria in recent years, Peru remains one of four countries in South America with endemic malaria where the incidence is increasing. To continue progress towards decreasing malaria incidence in Loreto, it is vital to understand the knowledge, attitudes, and practices related to malaria of those living in this region that are at the highest risk of infection. Malaria elimination can only occur when these communities are involved and have the knowledge and resources to prevent, identify, and treat malaria cases. This exploratory case study provides an understanding of the Sucusari community’s location and culturally specific knowledge, attitudes, and practices related to malaria. The rich narratives and experiences shared by this community, highlight factors that contributed to the uptake of preventive behaviours, what barriers existed to eliminating malaria, and how these can be addressed through culturally resonant, community-based programming. While these results are from one of many communities in the Peruvian Amazon, important insights can be gleamed from shared narratives that can be of use for further investigations into the health experiences of similar communities that have limited access to health services.

## Supplementary Information

Supplementary material 1

## Data Availability

The qualitative data collected and analyzed during the current study are available from the corresponding author on reasonable request.
