# Supplementary material for: Knowledge, attitudes, and practices regarding malaria transmission and prevention in an indigenous Maijuna community: a qualitative study in the Peruvian Amazon
Source: Malar J. 2024 Oct 18;23:314. doi: 10.1186/s12936-024-05121-8 (PMC11490027; doi:10.1186/s12936-024-05121-8)
Supplement: Supplementary file 1 — Supplementary material 1 [file 12936_2024_5121_MOESM1_ESM.docx]

**Knowledge, Attitudes, and Practices Regarding Malaria Transmission and Prevention in an Indigenous Maijuna Community: A Qualitative Study in the Peruvian Amazon**

Kathryn M. Hogan^1^, MS, Michael Gilmore*^2^*, Ph.D., Graziella P. McCarron*^2^*, Ph.D., Brian M. Griffiths*^3^*, Ph.D., Jeffrey W. Koehler^4^, Ph.D., Guillermo A. García^5^, MSc, Michael E. von Fricken^6^, Ph.D.

*Author Affiliations*

*^1^ Department of International Health, Johns Hopkins Bloomberg School of Public Health, Baltimore, MD, USA*

*^2^ School of Integrative Studies, George Mason University, 4400 University Drive, Fairfax, VA, 22030, USA*

*^3^ The Earth Commons—Georgetown University’s Institute for Environment & Sustainability, 3700 O St. NW, Washington, D.C., USA*

*^4^Diagnostic Systems Division, US Army Medical Research Institute of Infectious Diseases, 1425 Porter Street, Fort Detrick, MD 21702*

*^5^MCD Global Health , 8403 Colesville Road, Suite 320, Silver Spring, MD, 20910 USA.*

*^6^ Department of Environmental & Global Health, One Health Center of Excellence, University of Florida,*

**Contact information**

Kathryn M. Hogan – [khogan11@jhmi.edu](mailto:kathrynhogan95@gmail.com)

Michael Gilmore – [mgilmor1@gmu.edu](mailto:mgilmor1@gmu.edu)

Graziella P. McCarron – [gmccarro@gmu.edu](mailto:gmccarro@gmu.edu)

Brian Griffiths – [bg742@georgetown.edu](mailto:bg742@georgetown.edu)

Jeffrey W. Koehler – [jeffrey.w.koehler4.civ@mail.mil](mailto:jeffrey.w.koehler4.civ@mail.mil)

Guillermo A. García – [ggarcia@mcd.org](mailto:ggarcia@mcd.org)

Michael E. von Fricken – [mvonf@ufl.edu](mailto:mvonf@ufl.edu)

*Correspondence to: Michael von Fricken, Associate Professor & Director of the One Health Center of Excellence, Emerging Pathogens Institute, University of Florida, 2055 Mowry Rd, Gainesville, FL 32610 mvonf@ufl.edu

**Supplementary Information**

**Interview Guide (English)**

Sample questions translated from English to Spanish asked during individual interviews in the community of Sucusari. It is important to note that these questions served to generally structure the proposed interviews, but pertinent follow-up questions were asked based upon participant responses.

**Sociodemographics**

1. Name or pseudonym:
2. Date of interview:
3. GPS coordinates of household:
4. Gender:
5. Age:
6. Birthplace:
7. How long have you lived in Sucusari?:
8. Are you Maijuna?:
9. Do you speak any Indigenous languages? If so, what languages?:
10. Marital status:
11. Number of children:
12. Number of people in household:
13. Head of Household:
14. How many years did you go to school?:

**Interview Questions**

1. What are some of the challenges in this community?
   1. What are some of the health challenges in this community?
   2. Is malaria a challenge?
2. Can you please tell me a bit about malaria?
   1. How do you get malaria?
3. Have you had malaria?
   1. How many times have you had malaria?
   2. When was the first time you had malaria?
      1. How did know you had malaria?
      2. How did you feel when you had malaria this time?
      3. When you had malaria this time, did you visit the health post?
         1. If yes, where did you go?
         2. How long did you feel sick before going to the health post?
            1. Why?
         3. Did you take a malaria test?
            1. Where did you take the malaria test?
            2. Was it an RDT or a microscopy test?
            3. What did the health provider say or do?
            4. Did you get medicine?

Did you take it all?

Why/why not?

- - - - 1. If you did not go to the health post, why? What did you do?
  1. When was the last time you had malaria?
     1. How did know you had malaria?
     2. How did you feel when you had malaria this time?
     3. When you had malaria this time, did you visit the health post?
        1. If yes, where did you go?
        2. How long did you feel sick before going to the health post?
           1. Why?
        3. Did you take a malaria test?
           1. Where did you take the malaria test?
           2. Was it an RDT or a microscopy test?
           3. What did the health provider say or do?
           4. Did you get medicine?

Did you take it all?

Why/why not?

- - - - 1. If you did not go to the health post, why? What did you do?

1. Have your children had malaria?
   1. How many times have your children had malaria?
   2. Did they take a malaria test each time?
2. Have you ever thought you had malaria but the test said you didn’t have it?
   1. What do you think happened?
   2. Did your fever and chills go away?
3. Can you still work when you have malaria?
   1. If not, how long are you unable to work when you have malaria?
4. When your parents had malaria, how did they treat it? (*free-list)*
5. Are there traditional remedies for malaria that you know about or use?
6. Where did you learn the information that you know about malaria? Did someone teach you about it?
7. Is there a time of year where there is more malaria in the community? Why?
8. Does anyone get malaria more than others in the community? Why?
9. Since you’ve been in the community, have the number of malaria cases in the community changed? Why?
10. Can you prevent malaria?
    1. How?
    2. Do you do this? Why?
11. Do you own a bed net?
    1. Where did you get it?
    2. Does everyone in your house own one?
    3. Do you sleep under the bed net every night? Why?
    4. What time do you usually go to sleep? What time do you wake up?
    5. Does everyone in your family sleep under a bed net? Why?
    6. When you leave the community (to hunt, to go to city) do you still sleep under a bed net? Why?
    7. Are there any other times you don’t sleep under it?
    8. Does your bed net always stay up or do you hang it every night?
    9. Do you use your bed net for anything else?
    10. Has it ever gotten holes in it?
        1. Did you fix it?
    11. Is there anything you don’t like about the bed nets?
12. Has anyone come to community to talk about malaria?
    1. Who and when?
    2. What did they say?
    3. What did you think of what they said?
13. Have there been any community fumigations/spraying done in Sucusari?
    1. Who did this?
    2. Was it done just once? When?
    3. Where did they spray?
    4. Why was it done?
    5. Did it work?
14. Do you think sometime in the future people in Sucusari will stop getting malaria?
    1. Why?
    2. How?
15. Is there anything else you would like the share about malaria?

**Interview Guide (Spanish)**

Sample questions translated from English to Spanish asked during individual interviews in the community of Sucusari. It is important to note that these questions served to generally structure the proposed interviews, but pertinent follow-up questions were asked based upon participant responses.

**Sociodemographics**

1. ¿Cuál es tu nombre completo?
2. Fecha de la entrevista:
3. GPS coordinates of household:
4. Sexo:
5. Edad:
6. ¿Dónde naciste?:
7. ¿Para cuánto tiempo has vivido en Sucusari?:
8. ¿Eres Maijuna?
9. ¿Hablas un idioma indígena? ¿Cuál es?:
10. ¿Estás soltero/a o casado/a:
11. ¿Cuántos hijos tienes?
12. ¿Cuántas personas viven en tu casa?:
    1. ¿Cuántos adultos viven en tu casa?:
    2. ¿Cuántos muchachos viven en tu casa?
13. ¿Quién es la cabeza de la casa?:
14. ¿Cuántos años asististe a la escuela?:

**Interview Questions**

1. ¿Qué son algunos de los problemas de salud en la comunidad?
2. ¿Me puedes contar un poco de la malaria?
   1. ¿Dónde proviene la malaria?
3. ¿Has tenido malaria?
   1. ¿Cuántas veces has tenido malaria?
   2. ¿Cuándo era la primera vez que tenías la malaria?
      1. ¿Cómo sabías que era la malaria?
      2. ¿Cómo te sentías físicamente cuando tenías la malaria?
      3. ¿Cuándo tenías la malaria, ibas a una técnica de salud?
         1. ¿Si no, por qué? ¿Que hacías?
         2. ¿Dónde fuiste?
         3. ¿Para cuánto tiempo te sentías enfermo/a antes que llegabas a la posta?
            1. ¿Por qué?
      4. ¿Hacías la prueba de malaria?
         1. ¿Dónde hacías la prueba?
         2. ¿Era una prueba rápida o la lámina?
         3. ¿Qué decía la técnica?
         4. ¿Te daba medicina?
            1. ¿Tomabas todo?

¿Por qué no?

- - - 1. Si no ibas a la posta, ¿qué hacías?
  1. ¿Cuándo era la última vez que tenías la malaria?
     1. ¿Cómo sabías que era la malaria?
     2. ¿Cómo te sentías físicamente cuando tenías la malaria?
     3. ¿Cuándo tenías la malaria, ibas a una técnica de salud?
        1. ¿Si no, por qué? ¿Qué hacías?
        2. ¿Dónde ibas?
        3. ¿Para cuánto tiempo te sentías enfermo/a antes que llegabas a la posta?
           1. ¿Por qué?
     4. ¿Hacías la prueba de malaria?
        1. ¿Dónde hacías la prueba?
        2. ¿Era una prueba rápida o la lámina?
        3. ¿Qué decía la técnica?
        4. ¿Te daba medicina?
           1. ¿Tomabas todo?

¿Por qué/ Por qué no?

- - - 1. ¿Si tengas malaria de nuevo, irías a la posta de nuevo?
         1. ¿Por qué/Por qué no?

1. ¿Tus hijos han tenido la malaria?
   1. ¿Cuándo era la última vez que uno de tus hijos tenía malaria?
      1. ¿Qué año era?
      2. ¿Cómo sabías que era la malaria?
      3. ¿Cómo se sentía físicamente cuando tenía la malaria?
      4. ¿Hacia la prueba de malaria?
         1. ¿Dónde hacías la prueba?
         2. ¿Era una prueba rápida o la lámina?
      5. ¿Cuándo tenía la malaria, iban a una técnica de salud?
         1. ¿Si no, por qué? ¿Qué hacían?
         2. ¿Dónde iban?
         3. ¿Para cuánto tiempo se sentía enfermo/a antes que llegaban a la posta?
         4. ¿Qué decía la técnica?
         5. ¿Te daba medicina?
            1. ¿Tomaba todo el/ella?

¿Por qué/ Por qué no?

1. ¿Alguna vez has pensado que tenías malaria, pero la prueba era negativa?
   1. ¿Cuántas veces?
   2. ¿Qué piensas pasaba?
   3. ¿Cuáles síntomas tenías durante esta enfermedad?
   4. ¿Cuál enfermedad tenías?
   5. ¿Como tratabas esta enfermedad?
2. ¿Todavía trabajas cuando tienes la malaria?
   1. ¿Cuándo tienes malaria, cuántos días demoran que no puedes trabajar?
3. ¿Cuándo tus padres han caído enfermo con la malaria, como la trataban ellos?
4. ¿Hay remedios tradicionales para la malaria?
   1. ¿Si hay, que son?
5. ¿Dónde aprendías la información que sabes sobre la malaria?
   1. ¿Cuándo la aprendiste?
6. ¿Malaria tiene una época en la comunidad?
   1. ¿Si hay, cuándo?
   2. ¿Por que es así?
7. ¿Hay alguna gente de la comunidad que caen enfermo con la malaria más que los demás en la comunidad?
   1. ¿Por que?
8. ¿Desde cuando llegaste en la comunidad, la cantidad de casos de malaria ha aumentado, ha bajado, o es igual?
   1. ¿Por que es así?
9. ¿Desde cuándo llegaste en la comunidad, alguien ha muerto de la malaria?
   1. ¿Cuándo era la última vez?
10. ¿Puedes protegerse de la malaria?
    1. ¿Como puedes protegerse de la malaria?

*Transition: Thank you for sharing about your experience with malaria in your family and the community, now I would like to learn a little bit about bed nets.*

1. ¿Tienes un mosquitero?
   1. ¿Cuándo empezaste a usar un mosquitero?
   2. ¿De dónde ha venido tu mosquitero?
   3. ¿Cuántos mosquiteros hay en tu casa?
   4. ¿Cada persona en tu casa duerme abajo de un mosquitero?
      1. ¿Por qué/Por qué no?
   5. ¿Hay veces cuando no usas un mosquitero?
   6. ¿Cuándo sales de la comunidad para andar en monte o a Mazan, por ejemplo, llevas tu mosquitero?
      1. ¿Por qué/Por qué no?
   7. ¿Hay algo que no te gusta de los mosquiteros que debe ser cambiado?
   8. ¿A qué hora duermes normalmente?
   9. ¿A qué hora despiertas normalmente?

*Transition: Thank you for sharing about bed nets, now I would like to learn a little bit about how malaria is talked about and treated in the community by outside organizations.*

1. ¿Ha venido gente a la comunidad para hablar sobre la malaria?
   1. ¿De cuál organización eran ellos?
   2. ¿Cuándo era la última vez?
   3. ¿Han venido varias veces?
   4. ¿Cuándo era la primera vez?
   5. ¿Que decían ellos?
   6. ¿Como te parecía lo que decían ellos?
2. ¿Había fumigado la comunidad alguna vez?
   1. ¿Cuál organizacion hacía esto?
   2. ¿Venían varias veces?
   3. ¿Cuándo era la última vez que han fumigado a la comunidad?
   4. ¿Cuándo era la primera vez que han fumigado a la comunidad?
   5. ¿Dónde fumigaban?
   6. ¿Cómo te parecía esto?
   7. ¿Por qué han hecho esto?
3. ¿Crees que, un tiempo en el futuro la malaria no va a existir en Sucusari?
   1. ¿Por qué/Por qué no?
4. ¿Hay algo más que quieres decir o compartir sobre la malaria en la comunidad?
